# Geant4-DNA Simulation of the Oxygen Depletion Coefficient and the Relative Change in OER in Proton-Irradiated Oxygenated Water

**DOI:** 10.3390/ijms27198716

**Published:** 2026-09-29

**Authors:** Chun Wu, Yifei Han, Fangjie Zhou, Yushan Zhou, Quan Tang

**Affiliations:** 1 School of Nuclear Science and Technology, University of South China, Hengyang 421001, China; 2014002037@usc.edu.cn (C.W.); 20252010110747@stu.usc.edu.cn (Y.H.); 20232010110733@stu.usc.edu.cn (F.Z.); 2 School of Nursing, University of South China, Hengyang 421001, China

**Keywords:** FLASH, UHDR, Geant4-DNA, oxygen depletion, intertrack interactions

## Abstract

FLASH radiotherapy delivers therapeutic doses at ultra-high dose rates and may reduce normal tissue injury while preserving tumor control, but its mechanisms remain unclear. Radiolytic oxygen depletion has been proposed as one contributor, yet the combined effects of initial oxygen conditions and the spatial distribution of protons on oxygen depletion remain insufficiently understood. Geant4-DNA was used to simulate water radiolysis induced by 100 MeV protons in oxygenated water. All primary protons were introduced simultaneously, representing an idealized limiting case of zero pulse duration. Initial dissolved oxygen concentration and source radius were varied to evaluate oxygen depletion coefficients and the temporal evolution of chemical species. Relative changes in oxygen enhancement ratio were estimated using the Grimes model. Oxygen depletion coefficients increased with initial dissolved oxygen concentration, whereas reducing the source radius and increasing proton spatial aggregation decreased net oxygen depletion per unit dose. Species evolution supported enhanced radical recombination or termination at the smaller source radius and competition with oxygen scavenging pathways. The relative change in OER was jointly determined by initial oxygen conditions, oxygen depletion coefficients, and absorbed dose, with greater sensitivity under hypoxic conditions. These findings show that initial oxygen conditions and proton spatial distribution jointly regulate microsecond-scale oxygen depletion and the associated relative change in OER.

## 1. Introduction

Radiotherapy has become the preferred clinical approach for various malignant tumors due to its non-invasive nature and broad applicability. However, radiation damage to normal tissues surrounding the target lesion during radiotherapy still limits the escalation of therapeutic doses and the improvement in patient prognosis. Compared to conventional dose rate irradiation in traditional radiotherapy, ultra-high dose rate (UHDR) irradiation can deliver the prescribed dose within a significantly shorter time frame. Preclinical studies have reported that this mode of delivery can preserve tumor control while reducing damage to normal tissues, a phenomenon commonly referred to as the FLASH effect [1,2,3]. In recent years, in vitro cell and animal studies have shown that different radiation types, including X-rays, electrons, protons, and heavy ions, can induce FLASH-related biological responses under UHDR conditions [4,5,6,7,8]. However, the underlying mechanisms of the FLASH effect remain unclear. The required dose, dose rate, pulse structure, and tissue oxygenation conditions to trigger this effect are still under investigation.

Several mechanisms have been proposed to explain the mechanisms of the FLASH effect, including differences in DNA damage and repair, mitochondrial and metabolic protection, reduced inflammatory responses, oxygen depletion, and enhanced radical recombination [9,10]. Among these, the oxygen depletion hypothesis suggests that UHDR irradiation can rapidly deplete dissolved oxygen in the local microenvironment, thereby reducing the probability of fixing oxygen-dependent DNA damage and further influencing cellular or tissue radiosensitivity. This hypothesis links the early-stage water radiolysis chemical processes with subsequent biological damage. Oxygen concentration and dose-normalized oxygen depletion can also be investigated experimentally and computationally, making this hypothesis tractable to quantitative evaluation [11]. Nevertheless, whether the net oxygen depletion induced by UHDR irradiation and the resulting changes in local tissue oxygenation can lead to definitive biological effects remains one of the central points of contention in the oxygen depletion hypothesis.

Early studies on oxygen depletion under UHDR irradiation primarily relied on direct measurements using polarographic electrodes or indirect inferences based on cell survival curves. For example, Howard et al. used ^60^Co to irradiate oxygenated water and reported an oxygen depletion coefficient of approximately 0.44 μM/Gy based on polarographic electrode measurement [12]. Epp et al. [13], Weiss et al. [14], and Ling et al. [15] estimated the oxygen depletion coefficient in cell-containing solutions. The coefficient, with values around 0.25–0.68 μM/Gy, was estimated from cell survival curves under nanosecond electron pulse irradiation [13,14,15]. With the development of phosphorescence quenching methods and the Oxyphor series of probes, further patterns in oxygen depletion coefficients have been discovered. Veljko Grilj et al. [16] used 6 MeV electrons and PtG4 probes to measure oxygen content changes in mouse skin, muscle, brain tissue, and implanted tumors during FLASH irradiation. Their results showed that the oxygen depletion coefficient was not constant but increased with rising tissue oxygen content over the low oxygen range and approached a plateau around 32 mmHg [16]. Meanwhile, using 10 MeV electron and PdG4 probe data, Petusseau et al. reported a positive association between the initial oxygen partial pressure and the oxygen depletion coefficient in mouse skin over a range of 0–70 mmHg [17]. In aqueous systems, Xu Cao et al. [18] measured the oxygen depletion coefficient in bovine serum albumin (BSA) solutions after 10 MeV electron irradiation by Oxyphor 2P probes. The results showed that as the initial oxygen partial pressure in the solution decreased from 22 mmHg to 9 mmHg, the oxygen depletion coefficient decreased from 0.37 μM/Gy to 0.34 μM/Gy [18]. These findings suggest that the oxygen depletion coefficient may be influenced by initial oxygen content, medium composition, and measurement systems.

A dependence on initial oxygenation has also been reported for proton irradiation at UHDR. El Khatib et al. [19,20,21] measured the oxygen depletion coefficients with PtG4 probes in different solution systems irradiated with 230 MeV proton. The results showed that in oxygenated water containing 5% BSA, the oxygen depletion coefficient increased with initial oxygen partial pressure within the range of 0–11.7 mmHg. In solutions containing small organic molecules such as glucose and glutathione, this range extended to 0–20 mmHg. Under intracellular conditions, the corresponding range extended to approximately 23.5 mmHg. Measurements performed with the same proton beam and probe system in mouse leg tissue and implanted tumors revealed a monotonic increase in the oxygen depletion coefficient over an initial oxygen partial pressure range of 0–40 mmHg [19,20,21]. These experimental data indicate that even with similar irradiation conditions, the oxygen depletion coefficient under UHDR irradiation is not constant but varies with environmental oxygen content and molecular composition.

In addition to initial oxygen content, the temporal and spatial clustering of incident particles under UHDR conditions may also alter the reaction network during the chemical stage of water radiolysis. In conventional dose rate irradiation, the time interval between adjacent incident particles is typically much longer than the characteristic timescale of the water radiolysis chemical stage, resulting in a low probability of inter-track reactions among reactive species generated along different particle tracks. In contrast, UHDR irradiation can shorten both the temporal and spatial intervals between incident particles. This closer proximity increases the probability that radicals generated from different particle tracks will encounter each other during diffusion, leading to recombination or termination reactions. Consequently, the yields and lifetimes of relevant chemical species are altered, which in turn modifies the oxygen depletion process [22]. However, the irradiation conditions required for such interactions between tracks to produce measurable changes in water radiolysis products remain disputed.

Monte Carlo simulations have produced differing conclusions regarding the importance of interactions between tracks. Thompson et al. used TOPAS to simulate water radiolysis induced by 100 MeV protons and found that these interactions did not substantially change the G values of species such as H_2_O_2_ within a dose range of 2–8 Gy [23]. In contrast, Baikalov et al. simulated 1 MeV electron irradiation in oxygenated water and reported higher G values of H_2_O_2_ and ∙OH at 58 Gy than at 12 Gy [24]. Larissa Derksen et al. further proposed that for source radii in the range of 1–100 nm, under irradiation by 100 MeV protons, the G-values of some radicals and recombination products increased with the number of incident particles [25]. Using TRAX-CHEM, Castelli et al. further suggested that spatial separations below 1 μm may be required before interactions between tracks substantially affect radiolysis chemistry [26].

Existing experimental and computational evidence therefore suggests that both initial oxygenation and the spatial distribution of particle tracks can affect the chemical network of water radiolysis under UHDR irradiation. However, their combined effects on the depletion of oxygen during proton irradiation have not been systematically quantified. To address this issue, this study employs Geant4-DNA to simulate the proton-induced water radiolysis process. A proton irradiation model is established using instantaneous incident particle delivery and the variation in oxygen depletion with initial oxygen concentration is analyzed under different source radii. An oxygen enhancement ratio (OER) model is employed as a characterization parameter to evaluate the potential impact of oxygen depletion on the oxygen enhancement effect. OER is conventionally defined as the ratio of the radiation dose required under hypoxic or anoxic conditions to that required under oxygenated conditions to achieve the same biological endpoint. In the model developed by Grimes et al., OER is expressed as a function of oxygen partial pressure [27]. This approach provides a quantitative framework for evaluating the coupled influence of initial oxygen concentration and proton track density on radiolytic oxygen depletion.

## 2. Results

### 2.1. Residual Oxygen Concentration at 1 μs and the Corresponding Oxygen Depletion Coefficients

**Figure 1:**
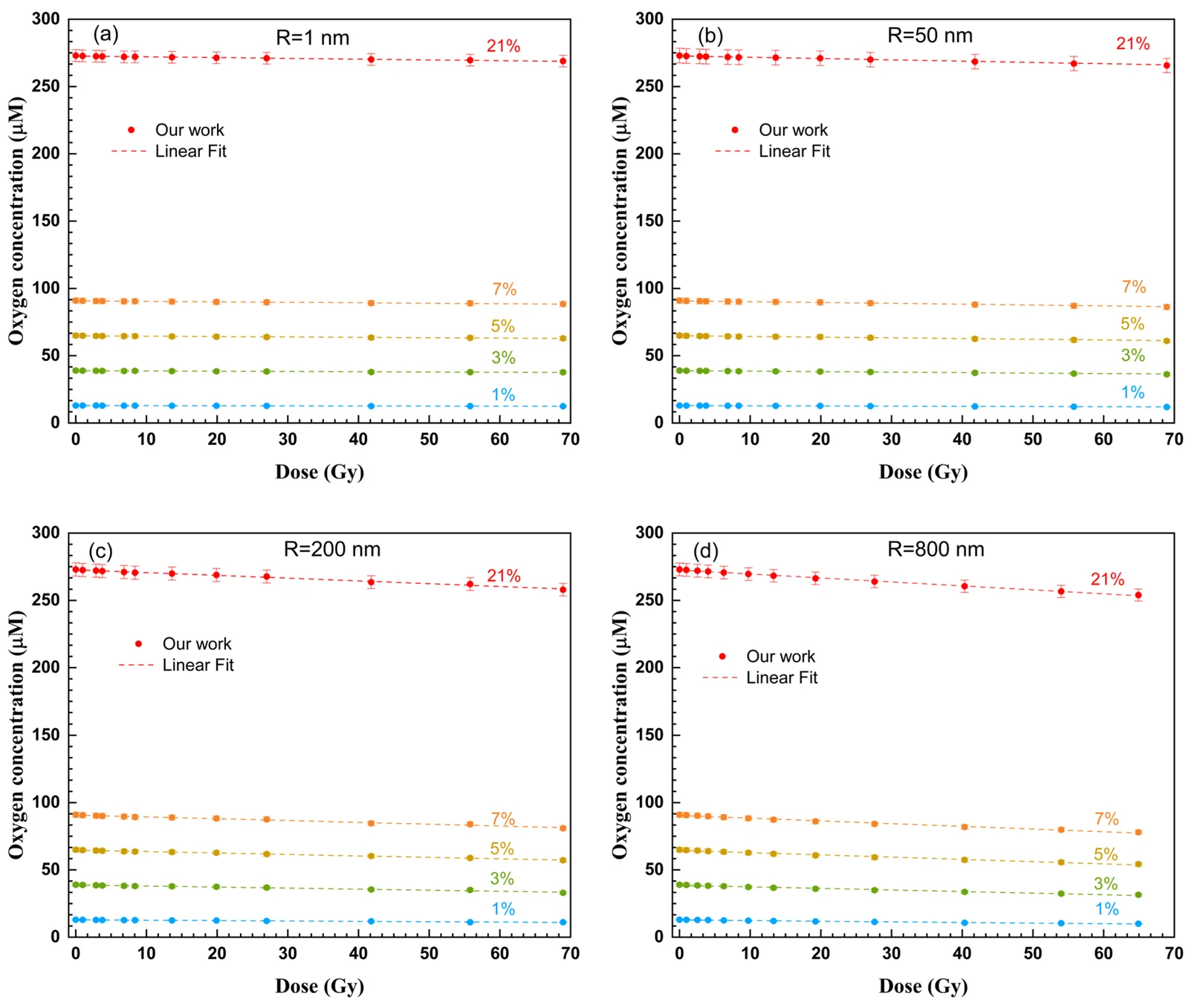
Residual oxygen concentration at 1 μs as a function of absorbed dose for different initial oxygen concentrations and source radii. (**a**) R = 1 nm, (**b**) R = 50 nm, (**c**) R = 200 nm and (**d**) R = 800 nm. Scatter points: simulation results from this work; dashed lines: linear fits.

Figure 1 presents the residual dissolved oxygen concentration at 1 μs as a function of absorbed dose for different initial oxygen fractions and source radii. Under all proton irradiation conditions examined in this study, the residual dissolved oxygen concentration decreased with increasing absorbed dose. The relationship was approximately linear over the investigated dose range, with coefficients of determination between 0.96 and 0.99.

The oxygen depletion coefficients calculated from the fitted slopes are summarized in Table 1. At a fixed source radius, the oxygen depletion coefficient increased with increasing initial dissolved oxygen concentration. Results reported by Boscolo et al. for oxygenated water irradiated with 1 MeV electrons and simulated using TRAX-CHEM were included as a qualitative external reference regarding the dependence of the oxygen depletion coefficient on the initial oxygen condition [28]. At the same initial oxygen concentration, the coefficient was consistently lower for R = 1 nm than for R = 800 nm. For example, at an initial oxygen fraction of 21%, reducing the source radius from 800 nm to 1 nm decreased the oxygen depletion coefficient from 0.29 to 0.056 μM/Gy. At all investigated initial oxygen concentrations, the oxygen depletion coefficient increased with increasing source radius, with the values obtained for *R* = 50 nm and *R* = 200 nm lying between those for *R* = 1 nm and *R* = 800 nm.

**Figure 2:**
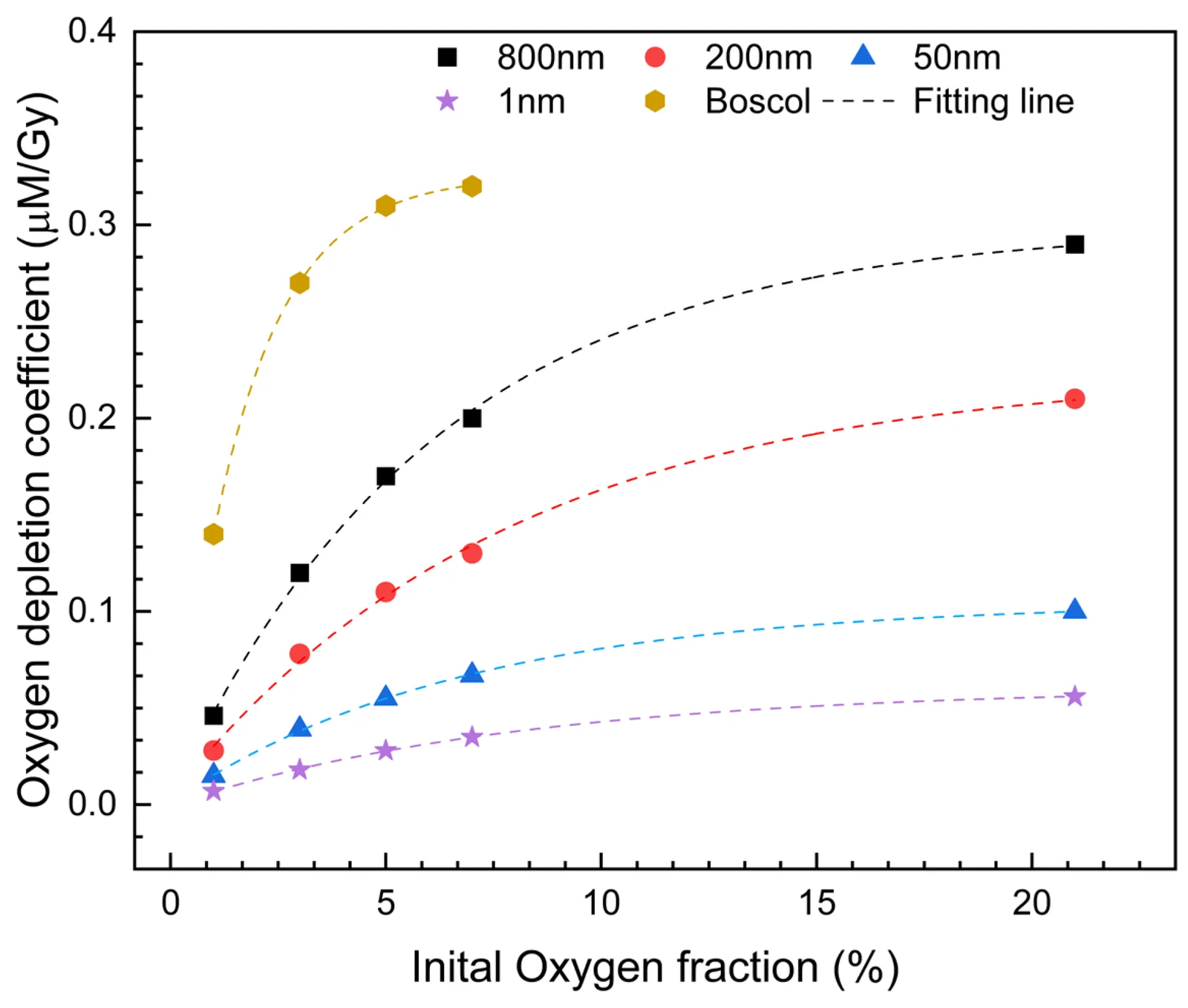
Variation in the oxygen depletion coefficient with initial oxygen concentration for different source radii. Data points represent the coefficients derived from fits to the residual oxygen concentration–dose relationship. Dashed lines show fits to Equation (1). Results for 1 MeV electron irradiation simulated by Boscolo et al. [28] are included for comparison.

Figure 2 further presents the dependence of the oxygen depletion coefficient on the initial oxygen fraction. For both source radii examined in this study and for the 1 MeV electron irradiation data reported by Boscolo et al. [28], the oxygen depletion coefficient increased with increasing initial oxygen fraction, although the relationship was nonlinear. Over the investigated concentration range, the exponential function given in Equation (1) provided a good empirical description of all three datasets, with R^2^ values greater than 0.99.

(1) $$D_{O_{2}}=a*\exp\left(-\frac{x}{b}\right)+a_{0}$$

$D_{O_{2}}$ represents the oxygen depletion coefficient, *x* denotes the initial oxygen fraction, *a*, *b* and *a*_0_ are fitting parameters (Table 2). The fitted curves increased rapidly at low oxygen fractions, whereas the rate of increase progressively decreased at higher oxygen fractions as the coefficient approached a plateau. The electron irradiation data exhibited a qualitatively similar dependence on the initial oxygen condition.

### 2.2. Temporal Evolution of Major Chemical Species Under Different Simulation Conditions

**Figure 3:**
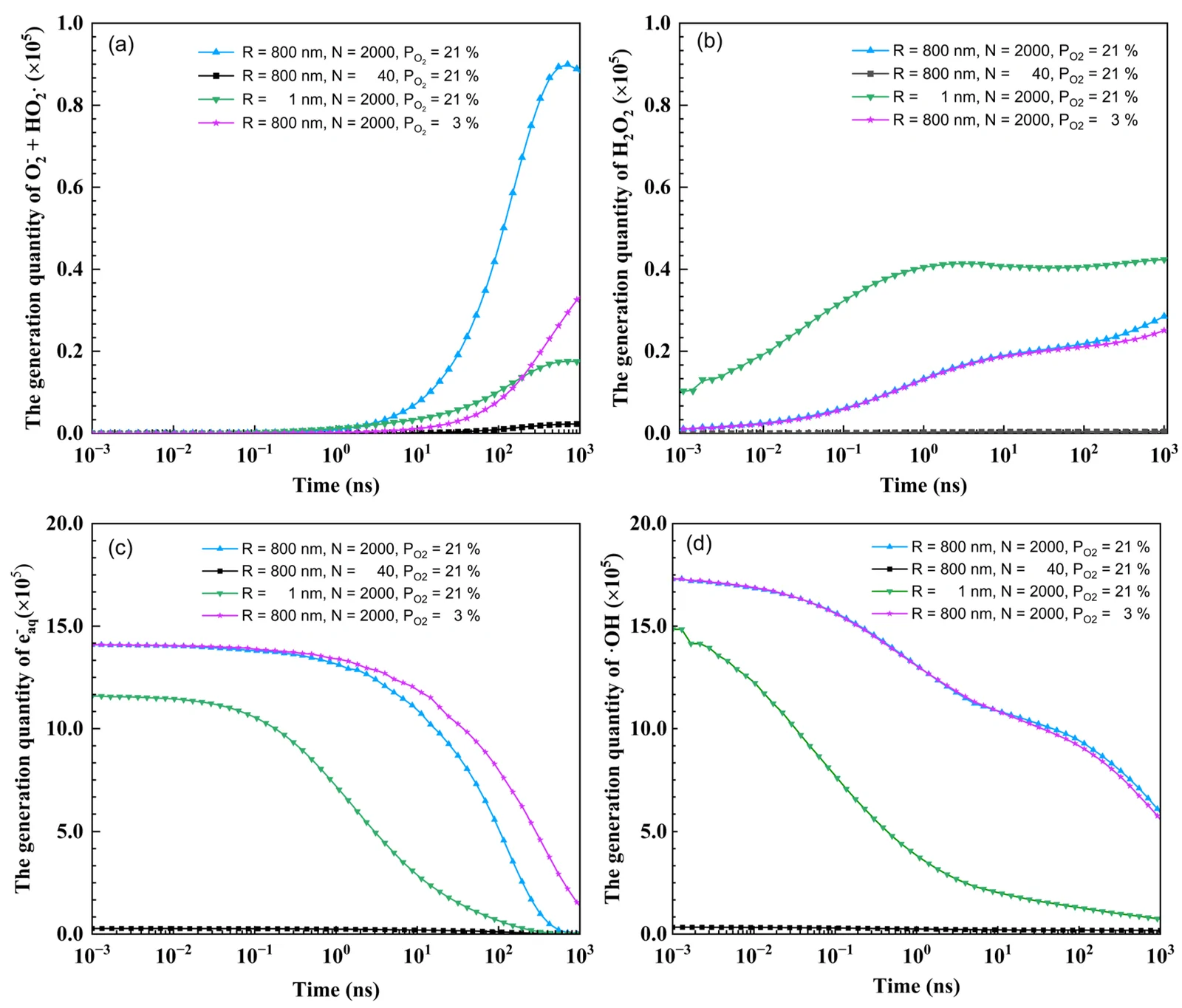
Time evolution of the yields of major chemical species under different numbers of incident protons, initial oxygen concentrations, and source radii. (**a**) ($O_{2}^{-}\cdot +{\mathrm{HO}}_{2}\cdot$), (**b**) H_2_O_2_; (**c**) $e_{\mathrm{aq}}^{-}$ and (**d**)·OH.

Based on the oxygen depletion results obtained across the investigated source radii, *R* = 1 nm and *R* = 800 nm were selected as representative conditions at the two ends of the radius range for the subsequent analysis of chemical species evolution. These two conditions correspond to the strongest and weakest spatial confinement investigated in this study, respectively. For the comparison between *R* = 1 nm and *R* = 800 nm, all irradiation and chemical conditions were identical except for the spatial distribution of the incident protons. Figure 3 shows the temporal evolution of the numbers of major chemical species from 10^−3^ to 10^3^ ns under different conditions of incident proton number, initial oxygen fraction, and source radius. Figure 3a,b present the temporal evolution of $O_{2}^{\cdot -}+HO_{2}^{\cdot }$ and H_2_O_2_, respectively, whereas Figure 3c,d depict the corresponding changes in $e_{\mathrm{aq}}^{-}$ and ∙OH. The condition with *N* = 2000, an absorbed dose of 68.93 Gy, an initial oxygen fraction of 21%, and *R* = 800 nm was used as the reference. In the other three conditions, the number of incident protons was reduced to, *N* = 40 the source radius was reduced to 1 nm, or the initial oxygen fraction was reduced to 3%. These comparisons were used to examine the effects of incident proton number, spatial distribution, and initial oxygen condition on the evolution of the chemical species.

(2) $$\begin{array}{cccc} & \cdot \mathrm{OH}+\cdot \mathrm{OH}\to H_{2}O_{2} & & \end{array}$$

(3) $$e_{\mathrm{aq}}^{-}+O_{2}\to O_{2}^{\cdot -}$$

(4) $$H^{\cdot }+O_{2}\to HO_{2}^{\cdot }$$

As shown in Equations (3) and (4), hydrated electrons and hydrogen atoms can react with dissolved oxygen to form $O_{2}^{\cdot -}$ and $HO_{2}^{\cdot }$, respectively. The combined abundance of these products therefore reflects the involvement of oxygen in these two scavenging pathways. Relative to the reference condition, reducing the number of incident protons, decreasing the source radius, or lowering the initial oxygen fraction all reduced the number of $O_{2}^{\cdot -}+HO_{2}^{\cdot }$. Under the reference condition, their combined number reached a maximum before 1 μs and subsequently decreased slightly. After the source radius was reduced to 1 nm, the number remained markedly lower than that obtained for R = 800 nm over the entire time range and gradually approached a stable level after several hundred nanoseconds. By contrast, when the initial oxygen fraction was reduced to 3%, the number of $O_{2}^{\cdot -}+HO_{2}^{\cdot }$ was still increasing at 1 μs and had not yet reached a maximum. The reduction in these oxygen-containing products after decreasing the source radius was consistent with the lower oxygen depletion coefficient reported in Table 1.

Figure 3b shows that, at the same incident proton number and initial oxygen fraction, the number of H_2_O_2_ was substantially higher for *R* = 1 nm than for *R* = 800 nm. Because H_2_O_2_ can be produced through hydroxyl radical recombination, as shown in Equation (2), this increase was consistent with a greater contribution from this reaction pathway. Figure 3c,d further illustrate that the numbers of $e_{\mathrm{aq}}^{-}$ and ∙*OH* decreased more rapidly for *R* = 1 nm. In particular, the number of $e_{\mathrm{aq}}^{-}$ fell to nearly zero within several hundred nanoseconds when *R* = 1 nm. When the initial oxygen fraction was reduced to 3%, the decrease in $e_{\mathrm{aq}}^{-}$ was slower, and a residual amount remained at 1 μs. In contrast, the temporal profiles of ∙OH were similar at initial oxygen fractions of 3% and 21%, indicating that the initial oxygen condition affected the temporal evolution of hydrated electrons and hydroxyl radicals differently.

Taken together, reducing the source radius from 800 to 1 nm decreased both the number of $O_{2}^{\cdot -}+HO_{2}^{\cdot }$ and the oxygen depletion coefficient, while increasing the number of H_2_O_2_ and accelerating the decline in hydrated electrons and hydroxyl radicals. These changes were consistent with a shift in the relative contributions of oxygen scavenging reactions and radical recombination, indicating that the spatial distribution of incident particles can influence the chemical reaction network and net oxygen depletion during the chemical stage.

### 2.3. Relative Changes in OER Estimated Using the Grimes Model

**Figure 4:**
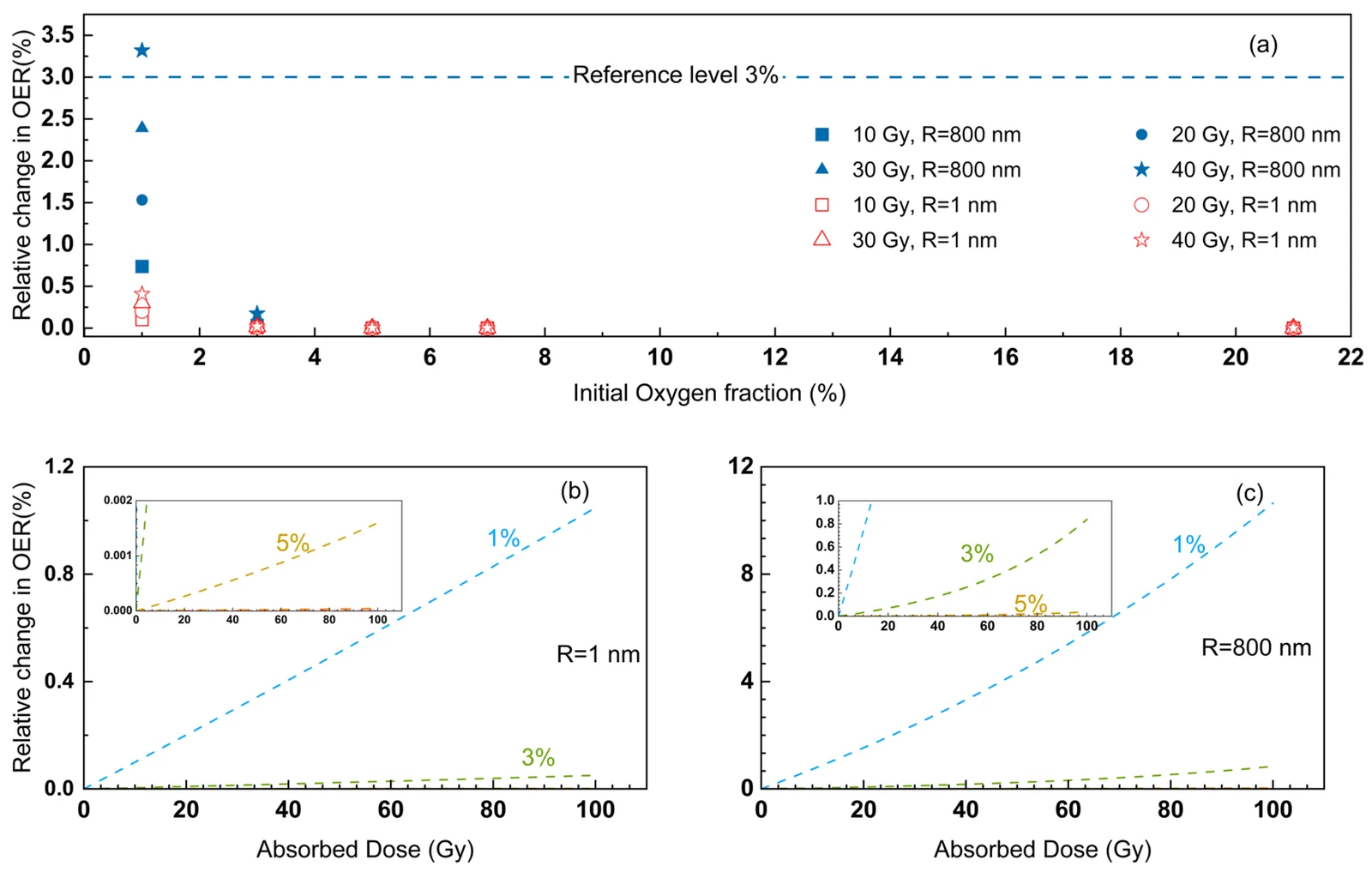
Relationships of ΔOER with initial oxygen fraction, absorbed dose, and source radius, estimated from the OER model. (**a**) ΔOER as a function of initial oxygen concentration under different doses and source radii. The dashed line indicates an illustrative reference level of ΔOER = 3%, adopted for comparison following Zhou et al. [29]. Subfigures (**b**,**c**) show ΔOER as a function of absorbed dose for a source radius of (**b**) 1 nm and (**c**) 800 nm.

Based on the OER model from Grimes et al. [27], the relative change in OER estimated from the initial oxygen fraction and the oxygen depletion calculated under each irradiation condition is illustrated in Figure 4. For both source radii, the relative change in OER increased with absorbed dose. However, the magnitude of this increment depended strongly on the initial oxygen fraction. At initial oxygen fractions of 3%, 5%, 7%, and 21%, the relative changes in OER are smaller than the reference level over the investigated dose range. In contrast, the largest changes were observed at an initial oxygen fraction of 1%.

At an initial oxygen fraction of 1%, increasing the absorbed dose from 10 to 40 Gy increased the relative change in OER from 0.7% to 3.3% for *R* = 800 nm. The corresponding value increased only from 0.1% to 0.4% for *R* = 1 nm. The smaller change obtained for *R* = 1 nm was consistent with the lower oxygen depletion coefficient reported for this source radius in Table 1.

Although the oxygen depletion coefficient increased with the initial oxygen fraction, a larger coefficient did not necessarily produce a larger relative change in OER. The greatest relative change was obtained under the lowest initial oxygen condition, whereas the changes remained limited at higher initial oxygen fractions. These results indicate that the relative change in OER was jointly determined by the initial oxygen condition and the extent of oxygen depletion included in the OER calculation.

## 3. Discussion

To clarify how the initial oxygen condition and the spatial distribution of incident particles influence oxygen depletion on the microsecond timescale during 100 MeV proton irradiation, Geant4-DNA was used to simulate proton-induced water radiolysis. The analysis examined the influence of the initial dissolved oxygen concentration and the source radius on the residual oxygen concentration and the oxygen depletion coefficient, along with the temporal evolution of major chemical species. Within each simulation event, all primary protons were introduced simultaneously at the initial time, corresponding to the limiting case of zero pulse duration. This setting eliminated temporal intervals between the primary protons and allowed the influence of their spatial distribution on chemical-stage reactions to be examined by varying the source radius at a fixed number of incident protons. This condition represents an idealized case of extreme temporal compression and does not reproduce the actual time structure of a proton FLASH beam.

The residual dissolved oxygen concentration at 1 μs was used to evaluate the net oxygen depletion induced by proton irradiation. The increase in the oxygen depletion coefficient with increasing initial dissolved oxygen concentration was consistent with the Monte Carlo results reported by Boscolo et al. [30] and Colliaux et al. [31]. Previous simulations also showed that the initial oxygen condition had only a limited effect on the G values of $e_{\mathrm{aq}}^{-}$ and H^∙^ before 1 ns. Therefore, under constant irradiation parameters, the key factor driving the differences in oxygen depletion coefficients was the variation in scavenging efficiency of dissolved oxygen against reducing species during the chemical stage, rather than any marked change in the early yields of $e_{\mathrm{aq}}^{-}$ and H^∙^.

At higher initial oxygen concentrations, dissolved oxygen can more rapidly scavenge $e_{\mathrm{aq}}^{-}$ and H^∙^, forming $O_{2}^{\cdot -}$ and $HO_{2}^{\cdot }$, respectively, and thereby increasing the contribution of reactions involving oxygen. As the initial oxygen concentration decreases, the effective reaction rate between oxygen and reducing radiolytic species also declines. Consequently, these reducing species can persist for longer periods or participate in other competing reactions, leading to less net oxygen depletion per unit dose.

The reaction between $e_{\mathrm{aq}}^{-}$ and O_2_ was used to examine this dependence quantitatively. Following the analytical approach proposed by Smith et al. [32], the bimolecular rate constant was set to $k_{O_{2}}=2.3\times 10^{10}M^{-1}s^{-1}$. When the dissolved oxygen concentration can be approximated as constant over the analyzed interval, the reaction can be expressed using pseudo-first-order kinetics:

(5) $$\frac{d[e_{\mathrm{aq}}^{-}]}{dt}=-k_{O_{2}}\left[O_{2}\right]\left[e_{\mathrm{aq}}^{-}\right]=-k_{\mathrm{obs}}\left[e_{\mathrm{aq}}^{-}\right],k_{\mathrm{obs}}\;=k_{O_{2}}[O_{2}]$$

The corresponding solution is

(6) $$[e_{\mathrm{aq}}^{-}](t)=[e_{\mathrm{aq}}^{-}{]}_{0}\exp\left(-\frac{t}{\tau }\right),\tau =\frac{1}{k_{\mathrm{obs}}}$$

Based on the initial dissolved oxygen concentrations listed in Table 3, the characteristic times for initial oxygen fractions of 21% and 3% were approximately 0.16 and 1.1 μs, respectively. These characteristic times represent the typical timescale over which $e_{\mathrm{aq}}^{-}$ is consumed through oxygen scavenging.

These timescales were consistent with the temporal evolution of $e_{\mathrm{aq}}^{-}$ shown in Figure 3c. At an initial oxygen fraction of 21%, the characteristic time was substantially shorter than 1 μs, indicating that $e_{\mathrm{aq}}^{-}$ could be rapidly scavenged by dissolved oxygen within the simulated time window. Accordingly, its number decreased to nearly zero within several hundred nanoseconds. When the initial oxygen fraction was reduced to 3%, the characteristic time increased to approximately 1.1 μs, which was comparable to the upper limit of the simulated time window, and a residual amount of $e_{\mathrm{aq}}^{-}$ remained at 1 μs.

Because oxygen scavenging of hydrated electrons generates $O_{2}^{\cdot -}$, this kinetic difference also partially explains the temporal evolution of $O_{2}^{\cdot -}+HO_{2}^{\cdot }$ shown in Figure 3a. At an initial oxygen fraction of 21%, the shorter characteristic time favored the rapid early accumulation of oxygen-containing products, whose combined number reached a maximum before 1 μs and then decreased slightly. This maximum reflects a change in the balance between their formation and subsequent consumption. When the initial oxygen fraction was reduced to 3%, the oxygen scavenging timescale increased, and the number of $O_{2}^{\cdot -}+HO_{2}^{\cdot }$ was still increasing at 1 μs while remaining substantially lower overall. Thus, the oxygen depletion coefficient was primarily governed by how the initial oxygen concentration modulated the rate at which reducing radiolytic species were consumed through oxygen scavenging reactions.

Under conditions of UHDR, a large number of particle tracks can be generated within a short time interval. The reactive species produced by different tracks may therefore coexist during diffusion, increasing the probability of chemical interactions between tracks. Alanazi et al. [33] used IONLYS-IRT to simulate the irradiation of air-saturated water with 300 MeV protons and examined the effect of increasing the number of simultaneously incident protons at a fixed source radius. Increasing the incident proton number enhanced interactions between tracks, reduced radical yields, increased molecular product yields and decreased oxygen consumption per unit deposited energy [33].

In the present study, a different parameter adjustment was used. The source radius was reduced from 800 to 1 nm while the number and energy of the incident protons and the initial oxygen condition were held constant. This increased the spatial density of the incident protons and the probability of chemical interactions between species generated by different tracks. Although the two studies modified different irradiation parameters, both showed that greater spatial aggregation of incident particles was associated with lower net oxygen depletion per unit dose. At an initial oxygen fraction of 21%, the oxygen depletion coefficients were 0.29 and 0.056 μM/Gy for *R* = 800 nm and *R* = 1 nm, respectively, corresponding to an approximately 5.2-fold difference. This result demonstrates that changing the source radius can substantially alter net oxygen depletion under otherwise identical simulation conditions.

To further quantify the differences in chemical-species evolution over the complete observation window, the number–time curves in Figure 3 were numerically integrated from 0 to 1 μs. The resulting time-integrated abundance reflects both the number of a species and the duration of its presence within the selected time window. For $O_{2}^{\cdot -}+HO_{2}^{\cdot }$, the time-integrated abundances were 7.01 × 10^7^ which was about 5 times the value of 1.40 × 10^7^ obtained for *R* = 1 nm. This ratio was close to the difference between the corresponding oxygen depletion coefficients, indicating that decreasing the source radius reduced both the formation of these oxygen-containing products and their persistence within the microsecond time window. By contrast, the time-integrated abundance of H_2_O_2_ increased from 2.53 × 10^7^ to 4.16 × 10^7^ molecule·ns as the source radius decreased, representing an approximately 1.6-fold increase. Since hydroxyl radical recombination is an important source of H_2_O_2_ in the reaction network, this increase was consistent with a greater contribution from radical recombination at the smaller source radius. This interpretation is supported by our previous Geant4-DNA study, where the G value analysis with *N* = 1 as a single-track reference showed that stronger spatial confinement enhanced inter-track chemical coupling and radical recombination [34].

The temporal profiles in Figure 3c,d provide additional support for this interpretation. At the same incident proton number and initial oxygen fraction, the numbers of both $e_{\mathrm{aq}}^{-}$ and ∙OH decreased more rapidly for *R* = 1 nm than for *R* = 800 nm. The decrease in $e_{\mathrm{aq}}^{-}$ under the *R* = 1 nm condition was even more pronounced than that observed under the 3% initial oxygen condition. The rapid reduction in radical numbers at *R* = 1 nm was not accompanied by an increase in $O_{2}^{\cdot -}+HO_{2}^{\cdot }$. Instead, it coincided with fewer oxygen-containing products and more H_2_O_2_. Therefore, the rapid disappearance of $e_{\mathrm{aq}}^{-}$ and ∙OH at the smaller source radius cannot be attributed solely to enhanced oxygen scavenging and is more consistent with increased radical recombination or termination reactions that do not consume oxygen.

Taken together, the oxygen depletion coefficients, the time-integrated abundances of $O_{2}^{\cdot -}+HO_{2}^{\cdot }$ and H_2_O_2_, and the temporal evolution of $e_{\mathrm{aq}}^{-}$ and ∙OH support a competitive relationship between radical recombination or termination and oxygen scavenging pathways. Reducing the source radius increased the spatial density of the incident particles and may have promoted reactions between reactive species generated by different tracks. These reactions could consume reactive species that would otherwise enter oxygen-related pathways, thereby reducing their contribution to oxygen scavenging. The redistribution of reaction pathways is therefore a plausible explanation for the lower oxygen depletion coefficient obtained at the smaller source radius.

OER is commonly used to characterize the influence of oxygenation on radiation-induced biological effects. It is traditionally defined as the ratio of the dose required under hypoxic or anoxic conditions to that required under oxygenated conditions to achieve the same biological endpoint. In the present study, the model developed by Grimes et al. on the basis of the oxygen fixation hypothesis was used to express OER as a function of oxygen partial pressure [27]. The model links the physical interactions induced by ionizing radiation, the subsequent chemistry of water radiolysis, and the probability of oxygen-mediated fixation of radiation damage.

According to the Grimes model, the relative change in OER associated with oxygen depletion depends jointly on the initial oxygen partial pressure, the oxygen depletion coefficient, and the absorbed dose. Although the oxygen depletion coefficient increased with increasing initial dissolved oxygen concentration, the calculated relative change in OER decreased rapidly as the initial oxygen condition increased. This apparently opposite trend arises from the nonlinear dependence of OER on oxygen partial pressure. In the Grimes model, the sensitivity of OER to a change in oxygen partial pressure is proportional to exp(−*ϕp*). As the oxygen partial pressure increases, OER approaches the saturation region of the response curve and becomes progressively less sensitive to further changes in oxygen partial pressure. Thus, greater oxygen depletion per unit dose under relatively oxygen-rich conditions does not necessarily produce a larger relative change in OER.

In a dimensional analysis of the minimum average dose rate required for pulsed FLASH irradiation, Zhou et al. adopted a 3% relative change in cell radiosensitivity as the minimum observable change and used this value to estimate the corresponding oxygen depletion requirement [29]. Following that study, a 3% relative change in OER was used in the present analysis as a reference level for comparisons among simulation conditions. This value was used as a comparative reference rather than as a universal biological threshold. Using this reference level, the oxygen depletion required to produce a 3% relative reduction in OER was estimated under different initial oxygen conditions.

At an initial oxygen fraction of 5%, the initial dissolved oxygen concentration was approximately 65.5 μM and the corresponding OER was approximately 2.63. The system was therefore already close to the plateau region of the model. To produce a 3% relative reduction in OER, the oxygen partial pressure would need to decrease from approximately 38.2 to 11.6 mmHg, corresponding to a reduction in the dissolved oxygen concentration to approximately 20 μM. Approximately 45 μM dissolved oxygen would therefore need to be depleted. Because the oxygen depletion coefficient under this condition was on the order of 10^−1^ μM/Gy, such a reduction would require an absorbed dose of several hundred Gy. This dose is substantially higher than that delivered in a conventional proton radiotherapy fraction. Thus, under relatively high initial oxygen conditions, the oxygen depletion predicted within clinically relevant dose ranges is insufficient to produce a marked change in OER through water radiolysis alone.

At an initial oxygen fraction of 1%, the initial dissolved oxygen concentration was approximately 13.1 μM and the corresponding oxygen partial pressure was approximately 7.65 mmHg. Under this condition, the system lies within a region where OER is more sensitive to changes in oxygen partial pressure. A 3% relative reduction in OER would require the depletion of only about 1.8 μM dissolved oxygen. Using the oxygen depletion coefficient obtained for *R* = 800 nm, the required absorbed dose was estimated to be approximately 36 Gy, consistent with the pronounced increase in the relative change in OER observed in Figure 4 under this condition. This comparison indicates that the magnitude of the oxygen depletion coefficient alone does not determine the resulting change in OER. Instead, the effect is governed by the combined influence of the oxygen depletion magnitude and the position of the initial oxygen condition on the OER response curve.

The source radius further modulated this effect. At an initial oxygen fraction of 1%, the oxygen depletion coefficient for *R* = 1 nm was only approximately 14% of that obtained for *R* = 800 nm. Consequently, at the same absorbed dose, the smaller source radius produced a smaller decrease in oxygen partial pressure and a correspondingly weaker relative change in OER. Within the dose range investigated in this study, the relative change in OER under the *R* = 1 nm condition did not reach the 3% reference level, whereas this level was exceeded at higher doses for *R* = 800 nm. These results indicate that, under the idealized simulation conditions considered here, the reduction in the oxygen depletion coefficient associated with an increased spatial density of incident particles can attenuate the potential effect of radiation-induced oxygen depletion on OER.

Within the present water radiolysis model, these results suggest that radiolytic oxygen depletion produces only a limited relative change in the modeled OER under relatively oxygen-rich conditions within the investigated dose range, whereas the response becomes more pronounced under initially hypoxic conditions and remains dependent on the spatial distribution of the incident protons.

Beyond the idealized temporal delivery condition described above, two additional limitations concern the simulated medium and the application of the OER model. The simulated system consisted of homogeneous oxygenated water and did not include biological processes or responses. The calculated oxygen depletion coefficients therefore describe net oxygen consumption within the selected water-radiolysis reaction network and the 1 μs observation window. They should not be interpreted as direct estimates of oxygen depletion in cells or tissues, where biochemical reactions, oxygen transport, and spatial heterogeneity can alter oxygen dynamics.

The relative changes in OER were calculated using the model developed by Grimes et al. [27] on the basis of the oxygen fixation hypothesis. This model does not explicitly represent biological processes or directly predict cellular survival, tissue sparing, or the FLASH effect. Moreover, its parameters were mainly fitted to photon irradiation data obtained at energies ranging from 50 kV to 20 MV. The low LET of the 100 MeV protons used in this study provides a rationale for applying the model, but the resulting OER changes should be regarded as comparative numerical estimates rather than proton-specific biological predictions. The oxygen conditions used in the radiolysis simulations represented uniform dissolved oxygen concentrations in water, whereas the oxygen partial pressure relevant to OER generally describes the local oxygen environment associated with radiation damage fixation and is not equivalent to tissue-scale oxygenation. Henry’s law was used only to provide a model-level conversion between these quantities. Future studies should examine realistic proton pulse structures, establish OER relationships specific to proton irradiation, and clarify the correspondence between simulated dissolved oxygen concentrations and oxygenation at the tissue scale.

## 4. Materials and Methods

### 4.1. Geant4-DNA Simulation Framework

Water radiolysis converts the energy deposited by ionizing radiation into reactive chemical species through a sequence of physical, physicochemical, and chemical processes. During the physical stage, which occurs approximately from 10^−18^ to 10^−15^ s, incident particles interact with water molecules and induce excitation and ionization. During the physicochemical stage, approximately from 10^−15^ to 10^−12^ s, the excited and ionized water molecules undergo relaxation and dissociation, leading to the formation of species such as hydrated electrons, hydroxyl radicals, and hydrogen atoms. During the subsequent chemical stage, extending approximately from 10^−12^ to 10^−6^ s, these species diffuse and participate in the chemical reaction network. Water radiolysis induced by 100 MeV protons was simulated in oxygenated water using Geant4-DNA version 11.3 [35,36,37]. The physical stage was modeled using the G4EmDNAPhysics_option2 physics constructor. The physicochemical and chemical processes were simulated using the EmDNAChemistry chemistry list implemented in the Geant4-DNA UHDR example. For the oxygenated water system, the ChemOxygenWaterBuilder class was used to construct the reaction network involving dissolved oxygen, its derived species, and the products of water radiolysis.

### 4.2. Simulation Geometry, Irradiation Conditions, and Scoring Procedures

**Figure 5:**
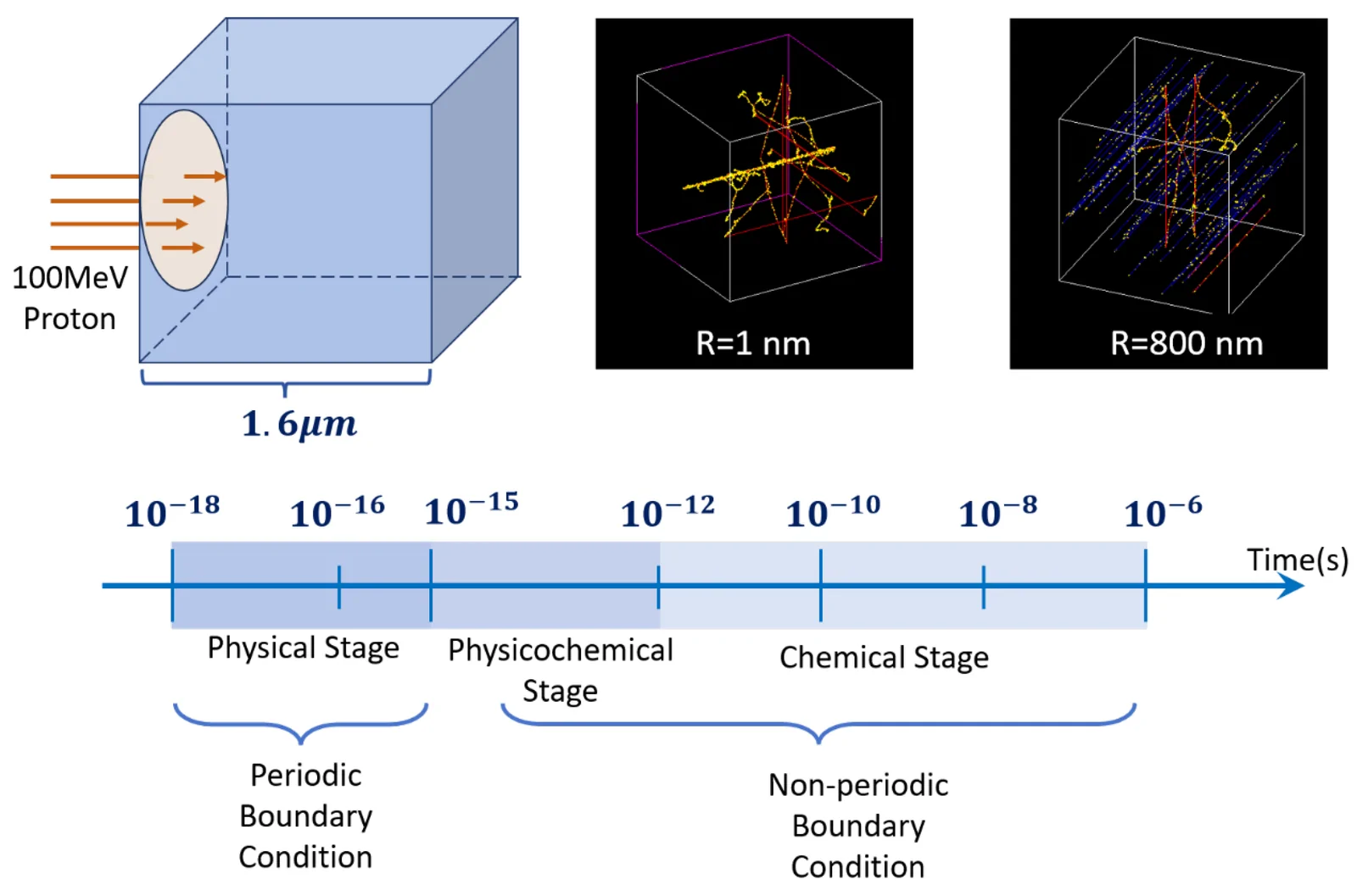
Schematic illustrating the geometry and time stages of the Geant4-DNA simulation. The setup consists of a cubic water phantom (1.6 μm side length) irradiated from the left by a 100 MeV proton beam emitted from a circular planar source. Simulations were performed with source radii of 1 nm, 50 nm, 200 nm and 800 nm. Periodic boundary conditions were employed exclusively in the physical stage, not in the chemical stage.

The simulation geometry is shown in Figure 5. The water phantom was a cube with a side length of 1.6 μm and was filled with oxygenated liquid water. During the physical stage, periodic boundary conditions were applied in all three Cartesian directions. This setting allowed secondary electrons and their progeny to continue their transport across the phantom boundaries and reduced boundary-related effects on energy deposition and absorbed dose. Our previous work showed that applying periodic boundary conditions during the physical stage did not cause an appreciable deviation in the calculated chemical yields when a small water volume was used to represent a larger aqueous medium [34]. Periodic boundary conditions were not applied during the physicochemical or chemical stages to avoid artificial effects on the diffusion and reactions of chemical species.

Each event represented an independent simulation realization containing a prescribed number of primary protons, denoted by *N*. The value of *N* controlled the total energy deposited and, consequently, the absorbed dose in the water volume. The number of simulated events was adjusted according to the irradiation condition to maintain sufficient statistical precision, and all reported quantities represent averages over the simulated events. The proton source was circular and was positioned in contact with the left surface of the water phantom. Four source radii (*R* = 1, 50, 200, and 800 nm) were investigated. In our previous study using the same Geant4-DNA framework, *R* = 1 nm represented a strongly spatially confined condition with pronounced inter-track chemical coupling, whereas *R* = 800 nm approached quasi-independent behavior [34]. In the present study, *R* = 50 nm and *R* = 200 nm were included as intermediate conditions to examine the evolution of oxygen depletion across the source radius range. The initial positions of the protons were sampled uniformly over the source surface, and the protons were directed perpendicular to the source plane. The proton energy was 100 MeV. According to the stopping power data in the NIST PSTAR database, the LET of 100 MeV protons in water with a density of 1 g/cm^3^ was approximately 0.73 keV/μm. The values of *N*, together with the corresponding energy deposition and absorbed dose in the 1.6 μm water phantom, are listed in Table 3. As an independent consistency check of the Geant4 energy deposition scoring, a total of 1000 independent simulations were run for a single proton (*N* = 1), each with a distinct random seed. The resulting mean deposited energy was 1.241 ± 0.169 keV per proton, where 0.169 keV represents the standard error of the mean. This result is statistically consistent with the approximate value of 1.168 keV derived from the LET and the 1.6 μm phantom thickness. All primary protons within an event were introduced simultaneously at the initial time, corresponding to the limiting case of zero pulse duration. This setting removed temporal intervals between primary protons and allowed the influence of their spatial distribution to be examined by changing the source radius at a fixed value of *N*.

The absorbed dose was calculated using the same scoring procedure described in our previous Geant4-DNA study [34]. Briefly, the energy deposited within the water phantom was accumulated over all particle-transport steps for each event. The absorbed dose was obtained from the total deposited energy and the mass of the scoring volume. Independent events were simulated using different random seeds and the reported dose represents the average over these events. The number of simulated events was adjusted according to the irradiation condition such that the relative statistical uncertainty of the absorbed dose did not exceed 5%.

Particle transport and energy deposition during the physical stage were simulated in liquid water, while, at the beginning of the chemical stage, dissolved oxygen was introduced as a uniformly distributed background species to model its reactions with the products of water radiolysis. The initial oxygen condition was specified using oxygen fractions of 1%, 3%, 5%, 7%, and 21% in the gas phase, covering conditions from low oxygen levels to air saturation. These fractions were converted into initial dissolved oxygen concentrations according to Henry’s law:

(7) $$C_{O_{2}}=KF_{O_{2}}P_{\mathrm{atm}}$$

where $C_{O_{2}}$ is the initial dissolved oxygen concentration, $F_{O_{2}}$ is the oxygen fraction in the gas phase, *P*_atm_ = 101,325 Pa is atmospheric pressure, and *K* = 1.30 × 10^−5^ mol m^−3^ Pa^−1^ is the Henry coefficient at 25 °C. The dissolved oxygen concentrations corresponding to the five initial oxygen fractions are listed in Table 3. The reactions involving oxygenated species and their corresponding rate constants are provided in Appendix A (Table A1).

The chemical stage was followed until 1 μs after the initial irradiation. At this time, the numbers of chemical species and the residual dissolved oxygen concentration in the water phantom were recorded. The residual dissolved oxygen concentration was obtained by averaging the values from independent simulation events as well. The number of events was adjusted so that its relative statistical uncertainty did not exceed 3%. Chemical species were counted using the G4MoleculeCounter class implemented in Geant4-DNA. The initialization and temporal tracking of dissolved oxygen were implemented using the Geant4-DNA UHDR example. Radiation-induced oxygen depletion was evaluated from the decrease in residual dissolved oxygen concentration with increasing absorbed dose. The oxygen depletion coefficient was obtained from the linear fit of residual dissolved oxygen concentration as a function of absorbed dose at 1 μs and was used to compare the net oxygen consumption per unit dose among different initial oxygen conditions and source radii.

### 4.3. Estimation of Relative Changes in OER

To further estimate the effect of oxygen depletion on oxygen enhancement, the model proposed by Grimes et al. [27] was used to calculate the oxygen enhancement ratio (OER). Based on the oxygen fixation hypothesis, this model links the physical interactions induced by ionizing radiation and the subsequent chemistry of water radiolysis to the probability that oxygen stabilizes DNA damage. OER is defined as the enhancement of cell killing under oxygenated conditions relative to that under anoxic conditions and is expressed as a function of oxygen partial pressure:

(8) $$\mathrm{OER}(p)=1+\frac{\Phi_{0}}{\Phi_{D}}\left[1-\exp(-\mathrm{\phi p})\right],\;\frac{\Phi_{0}}{\Phi_{D}}=1.63,\;\phi =0.26\;{\mathrm{mmHg}}^{-1}$$

where *p* is the oxygen partial pressure in mmHg. The ratio Φ_0_/Φ*_D_* represents the contribution of oxygen-dependent damage relative to damage under anoxic conditions, and *ϕ* is a fitting parameter that describes the saturation of OER with increasing oxygen partial pressure. Both parameters were obtained by Grimes et al. [27] through fitting experimental data.

The relative change in OER between the initial state and the state after oxygen depletion was calculated as

(9) $$\Delta \mathrm{OER}=\frac{\mathrm{OER}(p_{0})-\mathrm{OER}(p)}{\mathrm{OER}(p_{0})}\times 100\%$$

where *p*_0_ is the initial oxygen partial pressure and *p* is the oxygen partial pressure after irradiation. The latter was calculated as

*p* = *p*_0_ − Δ*p*, Δ*p* = *G_p_D* (10)

where *D* is the absorbed dose and *G_p_* is the oxygen depletion coefficient expressed in mmHg/Gy. The oxygen depletion coefficients obtained from the simulations in μM/Gy were converted to mmHg/Gy using Henry’s law at 25 °C before the OER calculation. The values calculated using Equation (9) are reported as percentages in Figure 4.

## 5. Conclusions

This study used Geant4-DNA to investigate how the initial oxygen condition and the spatial distribution of primary protons influence oxygen depletion on the microsecond timescale during proton irradiation of oxygenated water. All primary protons were introduced simultaneously within each event, representing an idealized limiting case of zero pulse duration rather than a realistic proton FLASH beam. The relative change in OER induced by oxygen depletion was also evaluated. The results indicate that radiolytic oxygen depletion is not governed by absorbed dose alone, but is jointly regulated by the initial dissolved oxygen concentration and the spatial distribution of the incident particles.

The initial oxygen condition influences net oxygen depletion primarily by altering the scavenging rate of reducing radiolytic species by dissolved oxygen. Higher initial oxygen concentrations favor oxygen-related reaction pathways, whereas lower oxygen concentrations prolong the persistence of reducing species and increase their probability of entering alternative reaction pathways. At the same time, greater spatial aggregation of primary protons enhances interactions among reactive species generated by different tracks. This promotes competition between radical recombination or termination reactions and oxygen scavenging pathways, thereby reducing net oxygen depletion per unit dose. The initial spatial distribution of incident particles and the resulting intertrack interactions are therefore important factors regulating reaction pathways and oxygen depletion efficiency during proton-induced water radiolysis.

Analysis based on the Grimes model further showed that the relative change in OER induced by oxygen depletion is jointly determined by the initial oxygen condition, the oxygen depletion coefficient, and the absorbed dose. The initial oxygen condition affects not only the magnitude of oxygen depletion but also whether the system lies within the oxygen-sensitive or saturation region of the OER response curve. Consequently, a larger oxygen depletion coefficient does not necessarily produce a more pronounced relative change in OER. Under hypoxic conditions, a relatively small change in oxygen concentration may induce a more substantial change in oxygen enhancement, whereas at higher oxygen levels, the response of OER to changes in oxygen partial pressure becomes progressively weaker.

Overall, this study establishes a quantitative link among the initial oxygen condition, the spatial distribution of primary protons, intertrack chemical interactions, microsecond-scale oxygen depletion, and the relative change in OER. The findings demonstrate that both the initial oxygen environment and the spatial distribution of incident particles should be considered when analyzing oxygen depletion under proton UHDR irradiation. These results provide a theoretical basis for further development of proton FLASH oxygen-depletion models incorporating realistic beam time structures, oxygen diffusion, and biochemical environments.

## Figures and Tables

**Table 1:** Oxygen depletion coefficients under different initial oxygen concentrations and source radii. Results for 1 MeV electron irradiation simulated by Boscolo et al. [28] are shown for comparison.

Initial Oxygen Content	21%	7%	5%	3%	1%
*R* = 800 nm	0.29	0.20	0.17	0.12	0.046
*R* = 200 nm	0.21	0.13	0.11	0.078	0.028
*R* = 50 nm	0.10	0.064	0.055	0.039	0.015
*R* = 1 nm	0.056	0.035	0.028	0.018	0.007
Boscolo et al. [28]	---	0.32	0.31	0.27	0.14

**Table 2:** Parameters of the exponential fit for the oxygen depletion coefficient as a function of initial oxygen concentration.

	a	b	*a*_0_
Boscolo et al. [28]	−0.30	1.65	0.33
R = 800 nm	−0.29	6.34	0.30
R = 200 nm	−0.22	7.02	0.22
R = 50 nm	−0.10	7.07	0.12
R = 1 nm	−0.06	8.11	0.06

**Table 3:** Key parameters used in the simulation.

Parameters	Values	Interpretation
Water phantom volume	1.6 × 1.6 × 1.6 μm	Liquid water with dissolved oxygen
Initial oxygen concentration	13.1 μM, 39.4 μM, 65.5 μM, 91.7 μM, 276.6 μM	Corresponding 1%, 3%, 5%, 7%, 21%
Proton energy	100 MeV	LET ≈ 0.73 keV/μm
Proton number	For calculating oxygen depletion coefficient 35 (0.97), 80 (2.87), 120 (3.75), 200 (6.83), 280 (8.39), 400 (13.61), 600 (19.9), 800 (27.00), 1200 (41.79), 1600 (55.79), 2000 (68.93)	The corresponding proton absorbed dose (Gy) in the water phantom is shown in parentheses.
	For calculating the time evolution of the yields of major chemical species 4, 2000	
Source radii	800 nm, 200 nm, 50 nm, 1 nm	

## Data Availability

The data presented in this study are available on request from the corresponding authors.

## Appendix A

Major reaction pathways of primary species in irradiated water and their associated reaction rate constants (k) employed during the chemical stage at 25 °C, taken from the ChemOxygenWaterBuilder class in Geant4-DNA-UHDR.

**Table A1:** Reactions involving oxygenated species and their corresponding rate constants.

Number	Reaction Pathways	k (10^10^·dm^3^·mol^−1^·s^−1^)
1	$e_{\mathrm{aq}}^{-}+O_{2}\to O_{2}^{-}$	2.3
2	$e_{\mathrm{aq}}^{-}+H\cdot \to H_{2}+{\mathrm{OH}}^{-}$	2.50
3	$e_{\mathrm{aq}}^{-}+e_{\mathrm{aq}}^{-}\to {\mathrm{OH}}^{-}+{\mathrm{OH}}^{-}+H_{2}$	0.636
4	$e_{\mathrm{aq}}^{-}+\cdot \mathrm{OH}\to {\mathrm{OH}}^{-}$	2.95
5	$e_{\mathrm{aq}}^{-}+H_{2}O_{2}\to {\mathrm{OH}}^{-}+\cdot \mathrm{OH}$	1.10
6	$e_{\mathrm{aq}}^{-}+H_{3}O^{+}\to H\cdot$	2.11
7	$e_{\mathrm{aq}}^{-}+O_{2}^{-}\cdot \to {\mathrm{OH}}^{-}+{\mathrm{OH}}^{-}+H_{2}O_{2}$	1.3
8	$e_{\mathrm{aq}}^{-}+{\mathrm{HO}}_{2}^{-}\to {\mathrm{OH}}^{-}+O^{-}$	0.351
9	$e_{\mathrm{aq}}^{-}+O^{-}\to {\mathrm{OH}}^{-}+{\mathrm{OH}}^{-}$	2.31
10	H_3_O^+^+OH^−^→No product	11.3
11	H_3_O^+^+O_3_→∙OH + O_2_	9.0
12	$H_{3}O^{+}+O_{2}^{-}\cdot \to {\mathrm{HO}}_{2}\cdot$	4.78
13	$H_{3}O^{+}+HO_{2}^{-}\to H_{2}O_{2}$	4.78
14	H_3_O^+^+O^−^→∙OH	4.78
15	H∙+H∙→H_2_	0.503
16	H∙+O_2_ → HO_2_∙	1.3
17	H∙+O^−^→OH^−^	2.00
18	H∙+HO_2_∙→H_2_O_2_	1.00
19	$H\cdot +O_{2}\to {\mathrm{HO}}_{2}^{-}$	1.00
20	$H\cdot +{\mathrm{OH}}^{-}\to e_{\mathrm{aq}}^{-}$	0.00251
21	H∙+H_2_O_2_→∙OH	0.0035
22	∙OH + H∙→No product	1.55
23	∙OH+∙OH → H_2_O_2_	0.55
24	$\cdot \mathrm{OH}+O_{2}^{-}\cdot \to O_{2}+{\mathrm{OH}}^{-}$	1.07
25	∙OH + OH^−^→O^−^	1.27
26	∙OH + HO_2_∙→O_2_	0.79
27	$\cdot \mathrm{OH}+HO_{2}^{-}\to {\mathrm{HO}}_{2}\cdot +{\mathrm{OH}}^{-}$	0.832
28	$\cdot \mathrm{OH}+O^{-}\to {\mathrm{HO}}_{2}^{-}$	0.1
29	$\cdot \mathrm{OH}+O_{3}^{-}\to O_{2}^{-}\cdot +{\mathrm{HO}}_{2}\cdot$	0.85
30	∙OH + H_2_O_2_ → HO_2_∙	0.00288
31	∙OH + H_2_ → H∙	0.00328
32	${\mathrm{OH}}^{-}+{\mathrm{HO}}_{2}\cdot \to O_{2}^{-}\cdot$	1.27
33	HO_2_∙+HO_2_∙→H_2_O_2_ + O_2_	0.000098
34	${\mathrm{HO}}_{2}\cdot +O_{2}^{-}\cdot \to {\mathrm{HO}}_{2}^{-}+O_{2}$	0.0097
35	${\mathrm{HO}}_{2}^{-}+O^{-}\to O_{2}^{-}\cdot +{\mathrm{OH}}^{-}$	0.035
36	$H_{2}O_{2}+{\mathrm{OH}}^{-}\to HO_{2}^{-}$	1.3
37	H_2_O_2_ + O^−^→HO_2_∙+OH^−^	0.0555
38	$O^{-}+O_{2}\to O_{3}^{-}$	0.37
39	O^−^+O^−^→H_2_O_2_ + OH^−^+OH^−^	0.01
40	$O^{-}+O_{3}^{-}\to O_{2}^{-}\cdot +O_{2}^{-}\cdot$	0.07
41	$O_{2}^{-}\cdot +O^{-}\to O_{2}+{\mathrm{OH}}^{-}+{\mathrm{OH}}^{-}$	0.06
42	$O_{2}^{-}\cdot +O_{2}^{-}\cdot \to H_{2}O_{2}+O_{2}+{\mathrm{OH}}^{-}+{\mathrm{OH}}^{-}$	0.000000001
